# Natural products as safeguards against monosodium glutamate-induced toxicity

**DOI:** 10.22038/IJBMS.2020.43060.10123

**Published:** 2020-04

**Authors:** Mohammad Mahdi Hajihasani, Vahid Soheili, Mohammad Reza Zirak, Amirhossein Sahebkar, Abolfazl Shakeri

**Affiliations:** 1 Department of Pharmaceutical Control, School of Pharmacy, Mashhad University of Medical Sciences, Mashhad, Iran; 2 Department of Pharmacodynamics and Toxicology, School of Pharmacy, Mashhad University of Medical Sciences, Mashhad, Iran; 3 Neurogenic Inflammation Research Center, Mashhad University of Medical Sciences, Mashhad, Iran; 4 Biotechnology Research Center, Pharmaceutical Technology Institute, Mashhad University of Medical Sciences, Mashhad, Iran; 5 School of Pharmacy, Mashhad University of Medical Sciences, Mashhad, Iran; 6 Department of Pharmacognosy, School of Pharmacy, Mashhad University of Medical Sciences, Mashhad, Iran

**Keywords:** Food industry, Herbal medicine, Monosodium glutamate, Neurotoxicity, Phytochemical

## Abstract

Monosodium glutamate is a sodium salt of a nonessential amino acid, L-glutamic acid, which is widely used in food industry. Glutamate plays an important role in principal brain functions including formation and stabilization of synapses, memory, cognition, learning, as well as cellular metabolism. However, ingestion of foodstuffs rich in monosodium glutamate can result in the outbreak of several health disorders such as neurotoxicity, hepatotoxicity, obesity and diabetes. The usage of medicinal plants and their natural products as a therapy against MSG used in food industry has been suggested to be protective. *Calendula officinalis, Curcuma longa*, Green Tea, *Ginkgo biloba *and vitamins are some of the main natural products with protective effect against mentioned monosodium glutamate toxicity through different mechanisms. This review provides a summary on the toxicity of monosodium glutamate and the protective effects of natural products against monosodium glutamate -induced toxicity.

## Introduction

**Figure 1:**
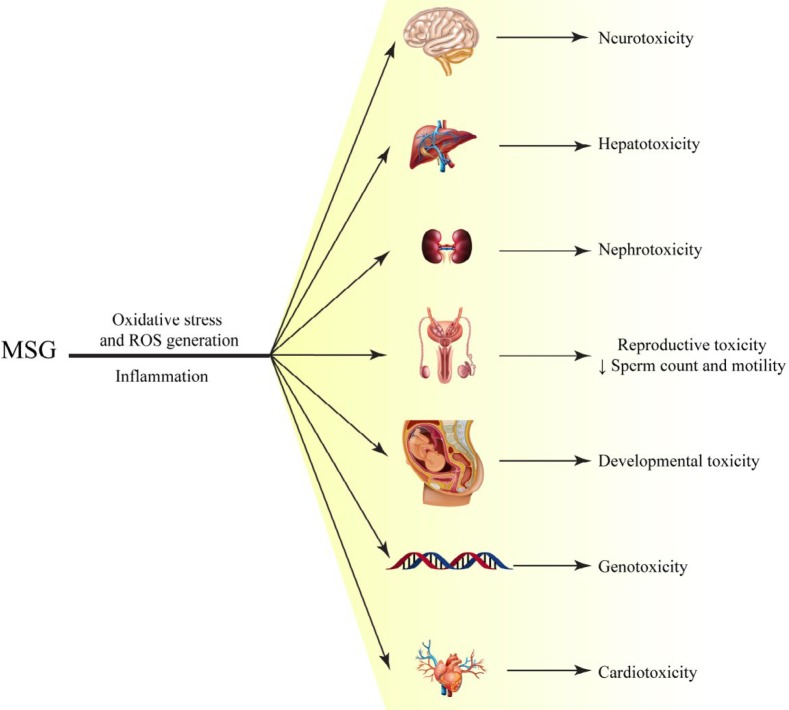
A schematic representation of Monosodium glutamate (MSG) toxicity and the organs may be affected by MSG

Food additives are extensively used in food industry in small quantities to improve the flavor, color, taste, appearance and texture of food (1). In food industry, the food flavoring agents are categorized to 25 classes, which contain about 230 different compounds (2). Among them, flavor enhancers are responsible for giving the food an umami, brothy and savory taste (3). Monosodium glutamate (MSG) is one of the most extensively used flavor enhancers and stabilizers in processed foods (4). The daily intake of glutamate depends on the region. The average intake of MSG has been estimated to be 0.3-1.0 g/day in developed countries (5). The average intake of MSG in UK was 0.58 g/day (6), 10.0 g in Germany (7) while it has recently been reported that in other European countries, the average daily intakes of glutamate is about 1.0 g/day. In Nigeria, an average intake of 0.56–1.00 g/day has been reported whereas in Asia it is higher with an intake of 1.1–1.6 g/day in Japan, 1.5–3.0 g/day in Taiwan and 1.6–2.3 g/day in South Korea (4, 8). Although the Food and Drug Administration (FDA) stated that MSG is a safe substance, several studies in animals have indicated negative effects after chronic consumption of MSG. These adverse effects have been shown in different organs such as thymus (9), brain (10), pancreas (11), testis (12), liver and kidney (13), and have been linked with several diseases including obesity, hypertension, headaches, asthma exacerbation, neurotoxic effects and detrimental effects on the reproductive organs (14). Figure 1 is a schematic representation of different organs that may be affected by MSG toxicity. Several natural products can exert protective effect against toxicity induced by MSG. In this review, the protective eﬀects of medicinal plants and natural products against MSG-induced toxicities are discussed.

**Search strategy**

This comprehensive review was performed by searching in Scopus, Web of Science, PubMed and Google Scholar to identify all published articles about the chemistry, toxicity of MSG, and protective effect of natural products against MSG from their inception up to August 2019. The search terms included “monosodium glutamate”, “toxicity”, “nephrotoxicity”, “hepatotoxicity”, “neurotoxicity”, “reproductive toxicity”, “oxidative stress”, “genotoxicity” and “natural products” in titles and abstracts.

**Chemistry of MSG**

**Figure 2:**
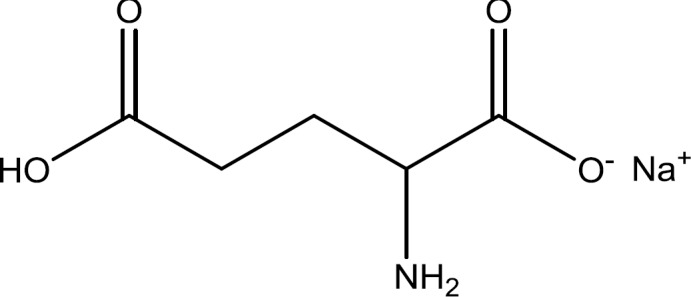
Chemical structure of Monosodium glutamate (MSG)

MSG (Figure 2) was first extracted from the seaweed *Laminaria japonica* and identified by the Japanese chemist Kikunae Ikeda in 1908 (15). It is a sodium salt of a nonessential amino acid known as L-glutamic acid, with chemical formula of C_5_H_8_NO_4_.Na, IUPAC-ID name of sodium 2-aminopentanedioate that specified by name of E621 in food industry (16). It is a white, odorless, and crystalline powder with a molecular mass of 169.11 g/mol and melting point of 232 ^°^C. It has a unique taste known as umami which is a savory, broth-like or meaty taste and once dissolves in aqueous solution; it will dissociate to form sodium and free glutamate. It is sparingly soluble in alcohol but the solubility in water is 385,000 mg/l at 25 ^°^C. MSG is also soluble in oil or organic solvents (17). It is a common glutamic acid salt which contains 78% glutamic acid, 22% sodium salt and also water (18). The major reason of using such an additive is that MSG has higher and more rapid dissolution performance against glutamic acid (19). 

**Pharmacokinetics**

Once ingested, MSG dissociates in the body into glutamate and sodium ions. Glutamate is one of the most common amino acid and abundant in the central nervous system (18). It is found in high concentrations in the regions of brain with essential role in mediating cognition incluan increase ding striatum, dentate gyrus of hippocampus and cerebral cortex (20). Since MSG does not require any enzyme activity to break it down, it is absorbed very quickly in our body which can spike the blood plasma levels of glutamate (21). Then, the absorbed glutamate is transported in the intestine lumen via sodium carboxylate transporter (NaDC-1) and excitatory amino acid transporter (EAAT-1), and circulated in the body through the bloodstream (22). There is a consensus that nearly whole ingested glutamate is metabolized in the intestine and most of its carbon skeleton is converted to CO_2_ or consumed for the intestinal production of amino acids such as glutathione, alanine, arginine, lactate and so others. Furthermore, most of its nitrogen is participated in the synthesis of proline, glutamine, amino acids of the urea cycle, and branched-chain amino acids. Remained little glutamate is absorbed into the portal vein which may be the reason for the low systemic concentration of glutamate (23). The plasma glutamate level peaked within 80 min, when it administered orally at dosage of 30 mg/kg every 20 min for 220 min, without any side effects (24). It was reported that excessive ingestion of MSG increased the concentration of glutamate and aspartate in plasma for 1–2 hr (25). Excessive daily intake of MSG causes an elevated plasma level of glutamate. The plasma levels of glutamate are depending on some factors including dose, concentration and age. For example, an increase in the concentration of MSG in neonatal rats (from 2 to 10%) leads to five-fold increase in plasma (26). It was found that doses of up to 1 g/kg of MSG do not significantly cross the blood–brain barrier (27). Furthermore, the glutamate levels in the brain are far higher than those in plasma of mice, rats, guinea pigs and rabbits (26). Actually, plasma glutamate concentrations are 50–100 *μ*M; while, they are 10,000–12,000 *μ*M in whole brain and just 0.5–2 *μ*M in extracellular fluids (28).

**Toxicity and side effects of MSG**

***Obesity and diabetes***

During recent decades, obesity has become a serious global health issue (29, 30). It plays an essential role in developing a wide range of human diseases such as dyslipidemia, diabetes, coronary heart disease, hypertension and cancer (30). Diet and lifestyle modification are suggested as two important aspects associated with reduced diabetes risk (31). It was suggested that uncontrolled use of food additives such as MSG can cause obesity (32). MSG has been used in several experimental models for inducing obesity which causes diabetes. Dietary MSG consumption is associated with obesity and overweight in healthy adults (33). The possible mechanisms involved in MSG-induced obesity may be the influence of MSG on energy balance by enhancing palatability through disrupting the hypothalamic signaling network of leptin action (33, 34). Administration of MSG enhances body weight, triacylglycerol, cholesterol, glucose, insulin, leptin and reduces high-density lipoprotein (HDL) levels in male Wistar rats (35). Injection of MSG (2 mg/g body weight) to the newborn male and female mice lead to increase blood glucose and diabetes (36). At the same dosage, MSG causes obesity and diabetes and moderates severe microvesicular fatty changes throughout the liver parenchyma in newborn mice (37). 

***Oxidative stress and hepatotoxicity***

**Figure 3.**
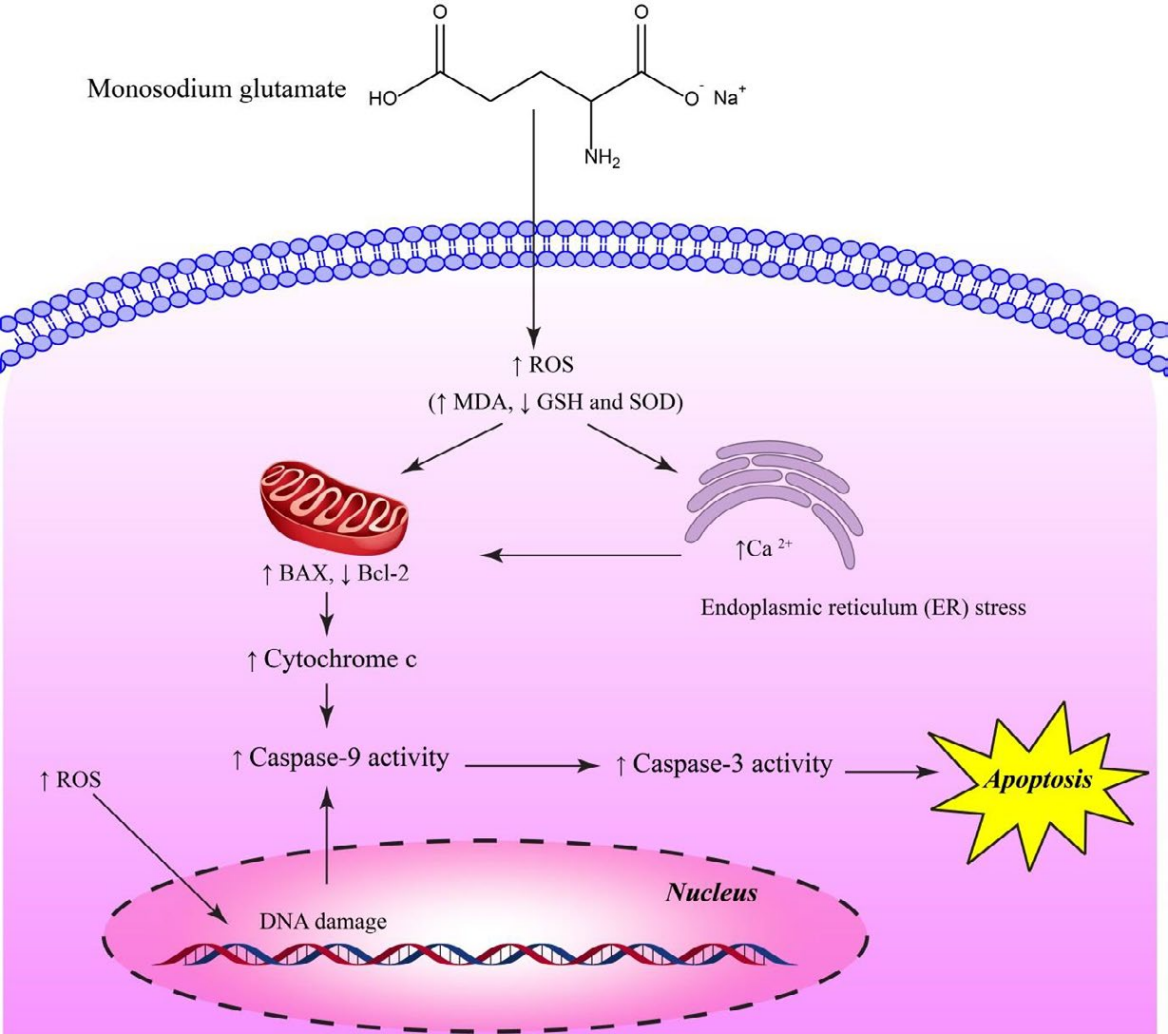
Molecular mechanism of monosodium glutamate (MSG)–induced cell death. As shown in the picture, MSG can activate intrinsic apoptosis pathway, leading cell death

The mechanism of action of MSG-induced damage to different organs such as the liver, brain, testis, and kidney is related to the induction of oxidative stress (38). Oxidative stress is a situation when intracellular levels of reactive oxygen species (ROS) are enhanced, which leads to the disruption of cellular metabolism and damage to lipids, carbohydrates, proteins and nucleic acids. Oxidative stress is associated with many human disorders including neurodegenerative diseases, diabetes, cardiovascular diseases, atherosclerosis, inflammatory bowel disease, osteoporosis and carcinogenesis (31, 39). Pavlovic *et al.* found that MSG increased rat thymocytes apoptosis *via* reducing Bcl-2 expression (40). The same authors also showed that prolonged administration of MSG to animals led to increased thymocytes apoptosis through oxidative stress (9). Molecular mechanism of MSG-induced cell death is shown in Figure 3. In another study on rats, dependent to the age, time of administration, and susceptibility of brain and spinal cord regions, MSG induced nociception and oxidative stress (41). Administration of MSG at doses more than 4 mg/g of body weight (bw) for adult male mice encouraged oxidative stress in erythrocytes by increasing lipid peroxidation (LPO, as a marker of oxidative stress) (42). Elshafey *et al.* (43) have demonstrated that MSG-induced hepatic toxicity through oxidative stress evidenced by increased lipid peroxidation, reduced antioxidant enzymes and fibrosis. Similarly, administration of MSG also has shown to induce oxidative stress through induction of lipid peroxidation, reduction of glutathione (GSH) and enhancement of the activities of superoxide dismutase (SOD), glutathione-s-transferase (GST), and catalase in the liver of the experimental animals which in turn, result in hepatotoxicity at a dose of 0.6 mg/g bw (44). Furthermore, administration of MSG induced oxidative stress in adult Wistar rats and causes some undesirable effects on the liver at higher doses (45). 

***Neurotoxicity***

MSG induces neurotoxicity through increasing LPO, oxidative stress and subsequent apoptosis and cholinergic dysfunction (46, 47). Although it is neurotoxic, manufacturers are using glutamic acid because it is cheap and do not want the public to know that (48). Its neurotoxicity associates with over-activation of excitatory amino acid receptors which causes enhanced intracellular calcium that triggers a cascade of enzymatic activities which resulting in cell death (49). Several studies also have demonstrated the neurotoxic effects of neonatal MSG administration (50, 51). MSG administration destroys neurons of the hypothalamus in rats which causes many metabolic abnormalities such as growth disturbances, self-mutilation, pseudo-obesity and hypogonadism (52-54). In addition, it is documented that MSG induces a neuroendocrine abnormalities leading to hypophagic which causes anxiogenic-like and depressive-like behaviors, metabolic dysfunctions, obesity, insulin resistance, changes in analgesic responses, glucose intolerance, and chronic inflammation (55-57). It is also found that MSG could be associated with other neurodegenerative illnesses such as amyotrophic lateral sclerosis, Alzheimer’s and Parkinson’s disease (58). 

Hippocampus, as one of the most vulnerable brain regions to glutamate-mediated excitotoxicity, has an important role in spatial learning and memory (59). Recently, it was found that rats exposed to low dose of MSG showed reduction in learning capabilities and short memory on forebrain in the hippocampus (48). Ali *et al.* have documented that low doses of MSG affects cognitive functions and has toxic effect during early childhood in which the blood-brain-barrier is highly permeable to large and small molecules (60). Such behavioral tests of animals exposed to MSG are also obtained by other studies (61, 62). In another study, exposed to MSG caused impairment of memory in rats associated to the inhibition of Na^+^, K^+^-ATPase activity in the hippocampus of rats (63). 

***Genotoxicity***

Several reports have been indicated the genotoxicicty of several flavour enhancers including MSG (64). It was also reported that MSG develops genotoxicity and cytotoxicity in the root tip cells of the plant *Allium cepa *(65). MSG presented both genotoxicity and mutagenicity in *Allium cepa* assay, that was manifested by chromosomal abnormalities and affected through mitotic index in root tips (66). *In vitro* studies have shown that MSG is genotoxic to the human peripheral blood lymphocytes (67). Furthermore, some other authors have demonstrated that MSG has genotoxic effects (66, 68, 69). The oral median lethal dose (LD_50_) of MSG in rats and mice was reported as 15 and 18 g/kg bw (70). Administration of MSG at doses of 20 and 40 mg/kg bw (equivalent to 1 and 2 g/person) for 2 months caused genotoxicity in rats’ palatal mucosa (71). ROS and oxidative stress are involved in MSG-induced cytotoxicity and genotoxicity, as indicated by increasing LPO in three major organs; liver, kidney and brain of rats (11). In contrast, some other researchers believed that MSG is not genotoxic (72, 73).

***Reproductive toxicity***

MSG induced testicular lesions in rats through oxidative stress. Administration of MSG at low and high doses caused alterations in the testis tissue of rats which may related to the male infertility in rats (74). Uses of MSG resulted in damage to the testes due to its effect in inducing oligospermia and glycogen accumulation in male Wistar rats (75). In another study, oral administration of MSG caused reduced levels of serum testosterone and decrease in the cauda epididymal sperm reserves in young male rats as well as adult ones (76). Several observed abnormalities in both microarchitecture of testis and semen characteristics has associated with using MSG administration in Wistar rats which could affect male fertility (77). A number of studied have mentioned that the ingestion of MSG could affect male fertility (78-80). It was reported that half lethal dose (LD_50_) of MSG in rats and mice is 15 and 18 g/kg BW respectively (70). Recently, European Food Safety Authority (EFSA) suggested the acceptable daily intake of MSG as 30 mg/kg BW.

**Protective effect of plants or plant-derived natural products**

***Calendula officinalis***

Shivasharan *et al.* (81) demonstrated the protective influence of the flower extract of *C. officinalis* against MSG-induced oxidative stress in addition to excitotoxic brain damage in rats. Orally administered of MSG to adult Wistar rats for 7 days resulted in increasing of oxidative and nitrative stress which evidenced by the reduced GSH, GST, total thiols, catalase activity and increased the MDA and nitrite levels in the brain tissues. It was previously reported that induction of apoptosis in the brain tissue resulted in neurotoxicity by MSG. When, animals were treated with the dosage of 100 and 200 mg/kg of the extract after one hour of MSG administration, it significantly reduced the oxidative stress through increased levels of CAT, GSH, TT, GST and reduced levels of LPO and nitrite. Since previous studies have demonstrated the antioxidant (82, 83) and anti-flammatory (84, 85) activities of *C. officinalis* flawers, they have suggested that the observed protective activity may relate to the antioxidant and anti-inflammatory activities of *C. officinalis *(81). Lima *et al.* (86) have also reported that 90 mg/kg extract of *C. officinalis* significantly reduced oxidative stress (increased GSH, SOD, CAT and reduced MDA) in rats. There are so many identified bioactive compounds in *C. officinalis *extract, which may responsible for aforementioned activities. Among them flavonoids such as quercetine (87) and the triterpenoids especially, the faradiol monoester (88), seem to play an important role as antioxidant and anti-inflammatory activities of *C. officinalis *extract, respectively.

***Cucurbita ficifolia***

Numerous experimental models have demonstrated that MSG in high doses can cause the increased body weight and fat mass (89). The increased body weight by MSG may be due to an increase in energy intake that subsequently lead to obesity (90), or interference with signaling systems regulating appetite centers, also upscaling food consumption led to weight gain (91). It is accepted that increased weight is associated with several diseases such as type 2 diabetes (T2D), reduced life expectancy, cardiovascular disease, psychological dysfunction and hypertension (92). Recently natural medicine or plant extracts with lowering body weight activity has attracted much attention due to its fewer side effects than chemical pharmaceuticals. Previous studies in T2D patients have confirmed that *C. ficifolia* (cucurbitaceae) has hypoglycemic activity (93). In other hand, a recent study showed that the aqueous extract of the endocarp of fruits from *C. ficifolia *(pumpkin) decreased the body weight and inflammation in an obesity model induced by MSG in an obesity model mice (MSG-obese mice) through inhibiting the expression of tumor necrosis factor type alpha (TNF-α), interleukin-6 (IL-6), tumor necrosis factor receptor 2 (TNFR2), while it increased the protein levels of interferon-gamma (IFN-γ) and IL-10. Moreover, the extract enhanced the protein levels of IL-10 in lean mice in addition to IFN-c in both lean and MSG-obese mice (94). Several studies have suggested that plants belong to cucurbitaceae family have anti-adipogenic property (95).

***Curcuma longa***

It was well-documented that *C. longa*, especially its bioactive component curcumin have numerous health effects including neuroprotective and anticancer properties (96-102). Khalil and Khedr (103) showed that curcumin has a protective role against MSG-induced neurotoxicity in rats. Curcumin treatment considerably attenuated both AChE activity and TNFα in MSG-treated rats. They suggested that anti-inﬂammatory activities of curcumin may explain this neuroprotective action. In another study, Vucic and colleagues reported that treatment of rat thymocytes with curcumin decreased MSG-induced apoptosis and ROS production, restored MMP and upregulated the Bcl-2/Bax protein ratio (104). In addition, they proposed that inhibition of PI3K/Akt signaling pathway in MSG-induced apoptosis was the main mechanism of anti-apoptotic effects of curcumin. Finally, the protective effects of curcumin on MSG-induced reproductive toxicity was shown by restoring testis weight and sperm count and decreasing the incidence of abnormal sperm in male rats (105). 

***Hibiscus sabdariffa***

*Hibiscus sabdariffa* belongs to the Malvaceae family, rich in several bioactive compounds such as flavonoids, anthocyanins, proanthocyanidins, polysaccharides and organic acids (106). Olaleye *et al.* identified also a verity of other compounds such as cardiac glycosides, saponins, alkaloids, and flavonoids in aqueous extract of *H. sabdariffa* (107)*.* It traditionally used to treat many diseases including liver disease, colds, hypertension, urinary tract infections, cholesterol-lowering and mutagenicity (106). In study of Gheller *et al.* aqueous extract of *H. sabdariffa *(at dose of 400 mg/kg/ day) exhibited considerable anti-mutagenic effects against MSG-induced DNA damage in male Wistar rats (108). The methanolic flower extract of another *Hibiscus *species*; H. tiliaceus* also has reported to have anti-mutagenic effects *in vivo* (109). Observed protection against mutagenic processes may be due to the presence of anthocyanins in the plants which act as potent antioxidants (110). Furtheremore, the anthocyanins extracted from *H. sabdariffa* have been reported as anti-carcinogenic and anti-mutagenic (111). Pre-clinical data have shown the anti-obesity effects of *H. sabdariffa *(112, 113). An *in vivo* study by Alarcon-Aguilar *et al.* (114) showed that orally administration of the aqueous extract of *H. sabdariffa* (120 mg/kg/day) for 60 days significantly reduced body weight gain and glycemia in MSG-obese mice.

***Green tea***

Obesity is known as an important risk factor for chronic morbidities such as cardiovascular diseases, some cancers (e.g. breast, colon and prostate), pulmonary and metabolic diseases (115). Both experimental and clinical studies have reported the anti-obesity effects of green tea (115, 116). MSG-induced obesity is widely used as an experimental model for investigation of obesity and its complications. Toxic effects of MSG on neurons of hypothalamus areas that control body mass and energy metabolism was reported as major mechanism of MSG-induced obesity (16). Study of Bártíková *et al.* showed significant anti-obesity effects of green tea. Oral administration of green tea extract (GTE) to obese mice resulted in reduction of food intake as well as level of insulin and leptin but did not significantly change the body weight (117). In addition, in another study, also it has reported that GTE could improve MSG-induced obesity and reduce insulin and leptin concentrations (118). Numerous studies have confirmed that MSG could cause damage to the ovaries of female rats, resulting in infertility (119, 120). MSG administration could cause oxidative stress in different organs which is associated with infertility in animal and *in vitro* models (121). Ali *et al.* have reported that GTE due to its potent antioxidant activity can protect ovarian against damages induced by MSG. They have concluded that the protection role of GTE against MSG is contribute to the ability of the green tea to trapping of ROS (122). Similar results were obtained by Yulianti *et al.* who reported that GTE (with 1.4 mg/day as an optimum dose) significantly increased the serum 17β-estradiol levels and Graafian follicles numbers in white rats exposed to MSG (123). In another study, an increased level of plasma total cholesterol, LDL-cholesterol and triglycerides was observed by oral administration MSG for 60 days. However, reduced gain body weight was observed in the groups treated with GTE at dose 1.5 ml/rat/day for 60 days (124). This observation is in accordance with other study finding that green tea catechins are associated to body weight regulation via inhibition of catechol *O*-methyl-transferase and phosphodiesterase which lead to thermogenesis, fat oxidation, and sparing fat free mass (125). Their results have also shown that GTE supplementation has the ability to normalize glucose levels and improve and ameliorate liver and kidney toxicity induced by MSG, restores the activity of antioxidant enzymes, and reduces the generation of ROS and lipid peroxidation (126). These results are in agreement with the findings reported in a previous study carried out by Hamza *et al.* (127), who noted that GTE conjugated with zinc oxide nanoparticles (ZnO/NPs) considerably recovers the hepatotoxicity developed by MSG through improving the liver enzyme activity (significantly reduced activities of LDH, ALT, AST, ALP as well as γ-GT in the serum) and the lipid profile (significantly decreased activities of LDH, ALT, AST, TC, γ-GT, TG, LDL-C, and VLDL-C).

***Mangifera indica L (Mango)***

Anthony and his colleague confirmed that MSG induced oxidative stress in brain of rats which evidenced by hypothalamic neuronal necrosis and degeneration of the brain histology. Therefore, they have studied the protective effect of mango (*Mangifera indica* L.) seed kernel against MSG toxicity in rats. Their study showed that high antioxidant potential of mango (increased the DPPH, total antioxidant capacity (TAC), and ferric reducing antioxidant power (FRAP) *in vitro*, besides increased catalase (CAT) and superoxide dismutase (SOD) activity and reduced MDA, glutathione peroxidase (GPx), glutathione (GSH) and uric acid (UA) *in vivo*) has a significant role in improving and regulating of the brain histology and serum antioxidant capacity of normal and MSG-intoxicated rats (128). The same author has already recorded similar results that demonstrated the modulation of MSG-induced toxicity in rats by mango seed kernel extract. Egbuonu and his coleagues have suggested that mentioned protective effect of the extract could be associated with the high vitamin C content of mango seed kernel which may enhance its antioxidant activity (129, 130). 

***Solanum lycopersicum (Tomato)***

As mentioned earlier, reproductive toxicity and infertility is one of the concerns of MSG usage in food industry. Given tomato to animals that exposed to MSG improved motility and morphology of spermatozoa in mice. Previously, the antioxidant effects of tomato content especially lycopene was suggested as the main mechanism (131). Lycopene, a naturally occurring bioactive compound in tomato is the best scavenger of the free radicals among carotenoids (132).

It was shown that lycopene had remarkable neuroprotective effects against MSG-induced cholinergic dysfunction, Bcl-2/Bax imbalance and neurotoxicity (47). Recently, Badawi has studied the protective effects of lycopene against MSG-induced nephrotoxicity *in vivo*. Orally administration of lycopene in a dose of 4 mg/kg/ day for 14 days in adult male albino rats showed that lycopene has protective effects against MSG-induced nephrotoxicity, through reduction of serum creatinine and blood urea nitrogen level, inhibition of apoptosis (decrease Bax, increase Bcl2), and prevention of kidney damage (133). Similar findings were obtained by Kadry (47) who proved that lycopene has protective effects against MSG-neurotoxicity through ameliorating oxidative stress (decrease LPO levels, SOD, GST, and catalase activities in addition to downregulating CAT and GST gene expression; increase GSH in the brain of rats) and apoptosis (decrease Bax, increase Bcl2), inducing improvement of the acetylcholinesterase activity, modulating antioxidant enzymes gene transcripts, and decreasing body weight. Numerous clinical trials have also confirmed that lycopene inhibited oxidative stress through preventing LDL oxidation (134, 135). In 2018, Lu *et al.* (136) have suggested that the inhibition of glutamate release is one of the potentially mechanism for neuroprotective actions of lycopene. They have confirmed that lycopene prevents glutamate release from rat cortical synaptosomes through inhibiting of presynaptic Ca^2+^ entry and PKC activity. 

***Walnut***

Walnut kernel mostly used as food but it also has been recorded as medicinal plant in traditional medicine (137). Study of Liang *et al.* showed that walnut meal extracts are rich in polyphenols such as glansreginin A, gallic acid and ellagic acid, significantly improved adverse effects of MSG on metabolic disorders including change in blood glucose, TG, TC, LDL-C, and insulin and liver dysfunction, as well as reduced the weight gain and fat accumulation (138). Previously, Rock *et al.* (139) have evaluated a study including one-hundred obese adults with two dietary strategies; a walnut-enriched diet group and a standard diet group for six months which resulted in reduced systolic blood pressure, triglycerides, total cholesterol, and low-density lipoprotein cholesterol (LDL-C), whereas their HDL-C, α-linolenic acid and linoleic acid were increased in the walnut-enriched diet group. They have suggested that the presence of γ-tocopherol and polyunsaturated fatty acids, such as α-linolenic and linoleic fatty acids are associated with the reduction in energy intake and weight loss. However, in another study, it was reported that gallic acid, as one of the main component of walnut, is able to reduce the body weight in rats, so it might be responsible for its weight-loss potential (140). The mechanism of action of the regulation of the body weight by gallic acid may explain in part via activating the AMP-activated protein kinase (AMPK) and improving mitochondrial function via the activation of peroxisome proliferator-activated receptor-γ coactivator1α (PGC1α) (141). 

***Zingiber officinale*** ***(Ginger)***

Ginger is traditionally used as spice since 2000 years ago. In addition, it is well known for its anti-emetic properties due to anti-5HT_3_-receptor effect, mostly used during pregnancy (142). Study of Hussein *et al.* showed that ginger had protective effects on MSG-induced neurotoxicity. Ginger reduced oxidative stress represented by decreasing MDA level as lipid peroxidation marker as well as increasing antioxidant enzymes activity. Moreover, treatment of rats with ginger (500 mg/kg orally) decreased MSG-induced elevated level of Na^+^ and Ca^+2^ in the brain while enhanced K^+^ concentration (143). Waggas (144) has studied the neuroprotective potential of the aqueous extract of ginger in MSG-induced neurotoxicity in male albino rats. He recorded that the chronic administration of 100 mg/kg of ginger extract ameliorated the toxic effects of MSG, evidenced by significant increase in epinephrine, norepinephrine, dopamine and serotonin (5-HT) content. In previous study made by Gomar *et al.* (145), the antioxidant and neuroprotective effect of ginger has confirmed. These beneficial effect of ginger may due to the presence of 6-gingerol and its derivatives such as 6-shogaol and 6-paradol, as well as zingerone which have potent antioxidant and anti-inflammatory activities (146-148).

***Ginkgo biloba***

*Ginkgo biloba* is well-known as neurodegenerative agent without side effects. Earliest Chinese medical record back to 2800 BC (149).* G. biloba *has been reported to have palliative effects on neuropathologic effects of MSG in male rats (150). *G. biloba *also prevented most of the damage caused by MSG in retinal pigmented cells. In addition, Elatrash *et al.* reported that *G. biloba* (80 mg/kg) had renoprotective and hepatoprotective activity exhibited by reduced serum level of urea, creatinine and uric acid (kidney function), alanine aminotransferase (ALT) and serum aspartate aminotransferase (AST) activity (liver function), and lipid peroxidation level of liver and kidney associated with MSG administration (151). Similar findings were obtained by Arafa (152), who stated that the administration of *G. biloba* extract in rats exposed to MSG, reduced serum activities of liver enzymes, kidney function, MDA and metallothionein (MT), while it increased glucose-6-phosphate dehydrogenase (G- 6-PD), GSH, and SOD activities. Furthermore, *G. biloba* has also shown protective effect on spinal cord neurons from MSG excitotoxicity and oxidative stress. This protection is via suppression of cytosolic phospholipase A_2_ (cPLA_2_) activation through ERK1/2 signaling pathway, which evidenced by significantly decreased in the expression of phosphorylated cPLA_2_ (p-cPLA_2_) and prostaglandin E_2_ (PGE_2_) release (153). It was found that the above mentioned beneficial effect of *G. biloba* may be associated with the presence of a sesquiterpene lactone compound named bilobalide (154). In fact, bilobalide was found as a potent neuroprotective *in vivo* through the reduction of ischemia-induced glutamate release, and reducing excitotoxicity (155). The same authors have demonstrated that bilobalide protected rats against glutamate-induced excitotoxicity neuronal death about 20–fold** (**EC_50 _= 5 μg/ml) better than *G. biloba* extract **(**EC_50 _= 100 μg/ml) through several mechanisms including anti-excitotoxicity, inhibition of ROS generation, scavenging of ROS, and regulation of mitochondrial gene expression (156).

***Algae***

Ovarian dysfunction that subsequently may results in female infertility rise some concern during last decades. Experimental studies reported adverse effects of MSG on female reproductive system showed by fetal growth retardation (157), neuroendocrine disorders in neonatal (158), hypothalamus and pituitary disorders (159), and changes in rat’s uterine tube (160). Algae such as *Spirulina platensis* and *Chlorella vulgaris*, as functional foods, has remarkable amount of components including carotenoids, micronutrient and essential amino acids that are necessary in human health (161). Regarding this, Abdel-Aziem and the colleagues showed that oral administration of female mice with *C. vulgaris* or *S. platensis* aqueous extracts, for 28 days, had protective effects against MSG-induced ovarian dysfunction represented by alleviate ovarian tissue histological change, sex hormones content and increased the ovarian enzymatic antioxidants (162). The protective effect of the aqueous extracts of both* S. platensis* and *C. vulgaris* against MSG toxicity including oxidative stress, genotoxicity and cell death pathway in male mice have studied (163). Administration of the aqueous extracts at dosage of 500 mg/kg bw showed hepatoprotective activity against MSG through enhancement of liver functions (MDA improvement, antioxidant activities, histochemical and histological alterations and DNA fragmentation), genotoxicity prevention, and apoptosis inhibition by means of advancing the Bcl-2 mRNA expression. They have suggested that the protective effect of both extracts against MSG-induced toxicities may be through the prevention of ROS because of the presence of natural antioxidants including minerals such as selenium and manganese, vitamins such as E and C, as well as β-carotene, tocopherol, C-phycocyanins and phenolic compounds (164).

***Vitamins***

*Vitamins C*

As vitamins and MSG may be present in human diet, it is, therefore, necessary to evaluate their interactions, in order to establish whether vitamins would exacerbate or ameliorate the adverse effects of MSG. Vitamin C (500 mg/kg) co-administered along with MSG for 45 days, showed hepatoprotective effect on the parenchymal architecture of the liver against MSG in rats through reducing cellular proliferation which evidenced by the low expression of ki-67 and tumor suppressor genes mutation (165). This anti-proliferative activity of vitamin C are mainly based on its extracellular action and induction of apoptosis, inducing cell cycle arrest and inhibition of expression of genes involved in protein synthesis (166, 167). Ashraf Waiz *et al.* (168) confirmed that administration of MSG at dose of 6 mg/g bw for 10 days induced hepatotoxicity and oxidative stress. Vitamin C (500 mg/kg) co-administered with MSG, significantly reduced the oxidative stress and hepatic toxicity with decreased LPO, ALT and AST activity and liver weight and reduced the hepatic activity of catalase. In another study, orally administration of vitamin C (100 mg/kg) attenuate the effect of MSG at doses of 2 and 4 mg/kg induced toxicity on the weight of testes and epididymis, sperm motility, sperm count as well as sperm head abnormality in rats (169). Vitamin C also inhibited the MSG-induced cytotoxicity in rat thymocytes through up-regulation of Bcl-2 protein expression (170). These results are in accordance with those concluded recently that MSG has cytotoxic effect on the germ cells in testicular tissue of adult rats, but vitamin C at dosage of 150 mg/kg due to its antioxidant properties has beneficial effect on reducing the cytotoxicity mediated by administration of MSG (171). Vitamin C has been demonstrated as protective agent against some other MSG-induced toxicities including histological changes in oviduct of rats (172), hepatotoxicity (173), sperm toxicity (169), Obesity (174), and neurobehavioral changes in periadolescent rats (175).

*Vitamin D*

It was found that vitamin D has protective effect against MSG-induced obese rats. Rats administrated with MSG showed the increase in the body weight, food and water intake. However, co-administration of vitamin D with MSG significantly suppresses body weight gain (176). Similar results were obtained by Elbassuoni *et al.* who reported that vitamin D and L-arginine have protective role in liver and kidney damage induced by MSG via inhibiting oxidative stress and decreasing food intake and body weight. The MSG-induced oxidative liver evidenced by increasing of renal MDA and decreasing of liver TAC and also kidney damage proved by increasing in the level of renal function markers, urea, and creatinine in rats. Concomitant vitamin D or L-Arginine administration with MSG protected liver and kidney against hepatic and renal injurious induced by MSG which is proved by decreasing in serum ALT and AST levels, and reducing serum urea and creatinine (the renal injury markers). Its molecular mechanisms may be the reduction of oxidative stress that was indicated by considerable decrease in MDA and remarkable increase in TAC in both hepatic and renal tissues (177). Furthermore, Nandan *et al.* (178) have reported that vitamin D might be helpful in the inhibition of the pre-and postnatal exposed MSG-induced steatohepatitis. They have also suggested that the diet rich in vitamin D might be beneficial in reducing the hepatic toxicity in the pregnant women consuming food containing MSG.

*Vitamin E*

Vitamin E is one of the most important antioxidants present in daily diet, which has protective effects against several human diseases (179). MSG at a dose of 0.6 mg/g bw made oxidative stress and hepatotoxicity in rats by induction of LPO, decreased the level of GSH, and increased the activities of GST, SOD and catalase in the liver of rats. Vitamin E (0.2 mg/g bw) co-administered with MSG (0.6 mg/g bw), ameliorated the LPO, increased the GSH level and decreased the hepatic SOD activities of GST, catalase, and reduced the ALT, AST and GGT activities in the serum (44). The administration of combined vitamin C and E prevented MSG-induced ovarian toxicity as shown by a significant decrease in the MDA levels and the quantity of atresia follicle and increased FSH level and quantity of primary follicles (180). Herbal oils such as sesame oil contain considerable amounts of vitamin E, polyunsaturated fatty acids and lignans, which are responsible for its high antioxidant capacity (181). Oral administration of sesame oil to rats reduced MSG-induced liver damages, showed by decreased in AST and ALT as well as oxidative stress indicators, and improved lipid profile (182). Furthermore, α-tocopherol, main constituent of vitamin E**,** at dosage of 200 mg/kg for 180 days protected nephrotoxicity caused by MSG which revealed by a significantly decreased in lipid peroxidation and oxidative stress (reduced MDA and conjugated dienes), improved renal function (decreased urea, uric acid, and creatinine) and increased antioxidant defense systems (SOD, CAT, GPx, GST, and GSH) (183). The same authors also have reported the protective role of α-tocopherol against MSG-induced cardiotoxicity in rats. Administration of MSG led to the oxidative stress which indicated by significant increasing in MDA and CD and decreasing in the activities of antioxidant defense systems; SOD, catalase, GSH, GSHpx and glutathione S-transferase, and increased activities of biochemical markers of cardiac dysfunction; creatine phosphokinase, aspartate transaminase, and lactate dehydrogenase. However, intake of α-tocopherol (200 mg/kg) was effective in reducing the cardiotoxicity of MSG (4 g/kg). In the other words, administration of α-tocopherol at dosage of 200 mg/kg for 180 days significantly reduced the MSG-induced oxidative stress and cardiac toxicities (184). α-Tocopherol as a potent scavenger of free radicals (185) may prevent development of oxidative stress related diseases possibly through antioxidant status mechanism via increasing the reduced glutathione and decrease the lipid peroxidation in the body (186). Therefore, the presence of vitamins especially C, D and E in foods containing MSG could be beneficial against MSG-induced toxicity.

***Other natural compounds***

In addition to the mentioned compounds above, there are other natural compounds that have been shown to possess protective effects against various MSG toxicities. These compounds are summarized in Table 1.

**Table 1:** Protective effects of some other plants or natural products against various Monosodium glutamate (MSG) toxicities

**Compound**	**Dose or Concentration**	**Experimental model**	**Target MSG-induced toxicity**	**Effects**	**References**
Tanshinone IIA	2.5-10 μM	Human neuroblastoma cell line SH-SY5Y	Neurotoxicity	↑SOD, CAT, Bcl-2, ↓ROS, MDA, Protein carbonyl content, Bax, cleaved caspase-3 apoptosis, JNK1/2, p38 MAPK	(187)
*Sida acuta* Leaf Extract	400 mg/kg/day, p.o., 14 days	Female rats	Neurotoxicity	↑ GSH,SOD and CAT activity↓ MDA level	(188)
Ethanolic extract of *Pongamia pinnata*	200 and 400 mg/kg/day, p.o., 7 days	Wistar albino rats	Neurotoxicity	↑ GSH,SOD and CAT activity↓ MDA level↓ Ca^+2^ and Na^+^ levels↑ level of K^+^	(189)
Naringenin	25, 50, 75 and 100 mg/L	Cultured hippocampal cells	Neurotoxicity	↑ Erk1/2 and Akt phosphorylation↓ Caspase-3	(190)
Methanolic and hydroalcoholic extract of *Solanum torvum*	100 and 300 mg/kg/day, p.o, 14 days	Swiss albino mice	Neurotoxicity	↑ GSH,SOD and CAT activity↓ MDA level	(191)
tetramethylpyrazine	10, 20 and 40 mg/kg/day, i.p, 10 days	Kunming mice	Neurotoxicity	↓ Expression NMDA receptor type 1 blocking Ca^2+^ influx	(192)
Aqueous extract of Rosemary	10 ml/kg/day, p.o., 42 days	Male albino rats	Neurotoxicity	↑ CAT activity, HDL level↓ MDA level, Cholesterol and LDL	(193)
Piperine	10 mg/kg/day, p.o., 14 days	Male Wistar rats	Neurotoxicity	↑ GSH content ↓ MDA level, glial fibrillary acidic protein (GFAP) and caspase-3	(194)
Butanolic extract of *Tinospora cordifolia*	10 and 20 μg/mL	Primary cerebellar cells	Neurotoxicity	↑ BCL-XL, MAP-2,GAP-43 and NF200↓ NF-κB, AP-1,iNOS, Cyclin D1 and IL-6	(195)
Ethanolic extract of garlic (*Allium sativum*)	12.5, 25, and 50 mg/kg/day, p.o., 10 days	Male Wistar rats	Neuronal excitotoxicity	Improved working memory performances	(196)
*Moringa oleifera* leaves extract	200 mg/kg/day, p.o., 28 days	Male albino rats	Hepatotoxicity and Genotoxicity	↑ GSH,SOD, GST and CAT activity↓ MDA level and hepatic enzymes (AST, ALT, ALP, and GGT)	(197)
Aqueous extract of *Trigonella foenum-graecum*	0.5 and 1 g/kg/day, p.o., 28 days	Neonatal Wistar rats	Dyslipidemia	↑ HDL, GSH,SOD, GST and CAT activity↓ MDA level, total cholesterol, triglyceridesand and hepatic enzymes (AST, ALT)	(198)
Aqueous extract of Qing brick tea	75, 150, and 300 mg/kg/day, p.o., 140 days	CD1 mice	Metabolic syndrome	↑ CAT, SOD and GPx activity↑ HO-1, Nrf2 and p-Akt expression ↓ MDA level, ROS and protein carbonylation↓ Cholesterol, triglyceride and FBS	(199)
Quercetin	75 mg/kg/day, i.p., 42 days	Male Wistar rats	Metabolic syndrome	↑ HDL, Total protein ↓ AST, ALT, ALP, LDH and Amylase↓ Cholesterol, triglyceride , LDL and VLDL, Creatinine	(35)
Quince (*Cydonia Oblonga*) leaf extract	500 mg/kg/day, p.o., 56 days	Male Wistar rats	Reproductive toxicity	↑ FSH and testosterone ↑ Epididymal sperm population and motility	(200)
*Centella asciatica*	100 and 200 mg/kg/day, 7 days	Female SpragueDawley rats	excitotoxicity	↑CAT, SOD ↓GSH, ROS, TBAR	(201)
Piper longum	300 mg/kg, 21 days	Male Wistar rats	Oxidative stress	↑ALT, AST ↓Lipid peroxides, GSH, Cholesterol, triacylglycerol	(202)
Garlic (*Allium sativum*)	100 mg/kg, 60 days	Wistar rats	Fibroid	↓ Cholesterol, estrogen, serum protein	(203)
Flaxseed Oil	1.2 ml/kg , 6 weeks	Male Wistar albino rats	Brain Injury	↑ Norepinephrine, Dopamine, Serotonin ↓ ALT, AST, Urea, Creatinine, MDA,	(204)
*Syzygium cumini*	0.5 or 1.0 g/kg/day, 8 weeks	Newborn male Wistar rats	Obesity	↑ Liver function ↓ Body weight, Fasting glucose, cholesterol, Triglycerides, Free fatty acids, ER Stress, Hepatic XBP-1s/PDI/MTP Axis	(205)
*Syzygium cumini*	500 mg/kg, 30 days	Newborn male Wistar rats	Metabolic syndrome	↓white adipose tissue, weight gain, Lee Index, triglyceride, cholesterol	(206)
Qing brick tea	75, 150 and 300 mg/kg, 20 weeks	Breeding CD1 mice	Metabolic syndrome	↑ SOD, GPx, CAT, GR, Nrf2/ HO-1, expression of p-AKT and GLUT4 ↓ MDA, ROS, protein carbonylation	(207)
coconut water	10 mL/kg b.w, 15 days	Male mice	Male infertility	↑ Sperm concentration, sperm motility, viable sperm	(208)
*Trigonella foenum-graecum* seeds	0.5 and 1.0 g/ kg b.w.	Neonatal male Wistar rats	Fat deposition and dyslipidemia	↓ Body weight, Lee’s index, white adipose tissue weights, adiposity index, glucose, insulin, leptin, LDL-C, VLDL-C, atherogenic index, coronary risk index, and homeostatic model assessment index	(209)
*Sapindus emariganatus*	200 mg/kg and 400 mg/kg, 28 days	wistar albino rats	Obesity	↓ Body weight, glucose, cholesterol, LDL-C, HDL-C, triglycerides, SGOT, SGPT, ALP	(210)
*Cedrus deodara*	100 and 200 mg/kg, p.o./day, 60 days.	Female Albino Wistar rats	Hyperlipidemic	↑ HDL ↓ body weight, serum glucose, cholesterol, triglyceride, LDL, VLDL	(174)
*Mimusops elengi*	100 and 200 mg/kg, 7 days	Adult female Wistar rats	Oxidative stress and excitotoxicity	↑ Locomotor activity, GSH, total thiols, GST, CAT ↓ LPO, brain nitrite	(211)
*Tinospora cordifolia*	20 μg/ml, 24 h	Primary cerebellar neurons	Excitotoxicity	↑ NF-κB, AP-1, HSP70, Mortalin ↓ MAP-2, GAP-43, NF200,Bcl-xL,	(195)

## Conclusion

MSG as a flavor enhancer is still being widely used in a variety of food preparations. Although this substance it is generally recognized as safe for limited use by FDA, numerous studies have recently indicated unwanted side effects of long-term consumption of MSG, making its safety and toxicity a controversial issue. However, a number of *in vitro* and *in vivo* animal models and even clinical trials have shown several potential health hazards of MSG particularly at high doses. There has been a consensus by many researchers that unusual effect of MSG extends to other tissues in the body. As discussed above, MSG can increase the risk of hypercholesterolemia, hypertriglyceridemia, obesity and diabetes. Furthermore, it can induce oxidative stress, hepatotoxicity and neurotoxicity. The aforementioned undesirable effects of MSG can be minimized by some medicinal plants and their constituents. This review provides some information on the protective role of medicinal plants and their active compounds against MSG-induced toxicity. Such natural products include curcumin from *Curcuma longa,* gingerols and shogaols from ginger (*Zingiber officinale)*, lycopene from tomato (*Solanum lycopersicum)*, rosmarinic acid from rosemary (*Rosmarinus officinalis*), piperine from pepper (*Piper nigrum*), and several vitamins which are suitable compounds to be added to food. These products have been shown to ameliorate health hazards of MSG* via* several mechanisms including enhancement of antioxidant status, inhibition of oxidative stress and reduction of apoptosis. For example, foods rich in lycopene, curcumin, quercetin and naringenin could inhibit oxidative stress-associated neuronal and liver damage in several related diseases. Based on the reviewed studies, it suggests that MSG should be eliminated from diet until its safety is re-examined. Moreover, it is imperative to do additional studies with more details to further understand the mechanisms underlying the serious health risks of MSG. 
